# The self-care profiles and its determinants among adults with hypertension in primary health care clinics in Selangor, Malaysia

**DOI:** 10.1371/journal.pone.0224649

**Published:** 2019-11-06

**Authors:** Hani Salim, Ping Yein Lee, Shariff Ghazali Sazlina, Siew Mooi Ching, Maliza Mawardi, Nurainul Hana Shamsuddin, Hanifatiyah Ali, Hanim Ismail Adibah, Ngiap Chuan Tan

**Affiliations:** 1 Department of Family Medicine, Faculty of Medicine and Health Sciences, Universiti Putra Malaysia, Serdang, Selangor, Malaysia; 2 SingHealth Polyclinics, Jalan Bukit Merah Connection One, Singapore; 3 SingHealth-Duke NUS Family Medicine Academic Clinical Programme, Jalan Bukit Merah Connection One, Singapore; Universidade Estadual Paulista Julio de Mesquita Filho, BRAZIL

## Abstract

**Introduction:**

Self-care has been shown to improve clinical outcome of hypertension. Gauging the level of self-care among patients with hypertension enables the design of their personalized care plans. This study aimed to determine the self-care profiles and its determinants among patients with hypertension in the Malaysian primary care setting.

**Methods:**

This was a cross sectional study conducted between 1 October 2016–30 April 2017 in three primary care clinics in the state of Selangor, Malaysia. All adults aged 18 years and above with hypertension for at least 6 months were recruited with a systematic random sampling of 1:2 ratio. The participants were assisted in the administration of the structured questionnaire, which included socio-demographic information, medical information and the Hypertension Self-Care Profile (HTN SCP) tool. Statistical analysis was done using SPSS version 20.0. Multiple linear regression was performed to determine the determinants for self-care.

**Results:**

The mean age of the participants was 59.5 (SD10.2) years old. There were more women (52.5%) and most were Malays (44.0%) follow by Chinese (34%) and Indians (21%). Majority (84.2%) had secondary or primary school level of education. A third (30.7%) had a family history of hypertension. The mean total HTN-SCP score was 124.2 (SD 22.8) out of 180. The significant determinants that influenced the HTN-SCP scores included being men (B-4.5, P-value0.008), Chinese ethnicity (B-14.7, P-value<0.001), primary level education/no formal school education level (B-15.7, P-value<0.001), secondary level education (B-9.2, P-value<0.001) and family history of hypertension (B 4.4, P-value 0.014).

**Conclusions:**

The overall hypertension self-care profile among patients in this multi-ethnic country was moderate. Being men, Chinese, lower education level and without family history of hypertension were associated with lower hypertension self-care profile score. Healthcare intervention programmes to address self-care should target this group of patients.

## Introduction

Hypertension is a common non-communicable disease (NCD) globally. As many as 22% of adults aged 18 years and above had raised blood pressure in 2014. [1] It was estimated that 17.5 million deaths were from cardiovascular related diseases in 2012, and three quarter of these deaths occurred in low- and middle-income countries.[1] According to the Malaysian National Health and Morbidity Survey 2015, the prevalence of hypertension among adults aged 18 years and above was 30.3% and the prevalence increases with age.[2] The prevalence in the rural areas (33.5%) were significantly higher than the urban areas (29%).[2]

Patients with hypertension are required to perform varying forms of self-care behaviours as part of their management. The major areas in hypertensive control that needed self-care attention includes medication taking and a number of lifestyle factors such as non-smoking, weight reduction, consumption of low-sodium and low-fat diet, increasing physical activity, moderation in alcohol consumption, self-monitoring of BP, regular doctor visits, and stress reduction.[3,4] Patients’ ability to manage themselves is correlated with better clinical outcomes, better blood pressure control, reduced frequency of medical consultations, and consumption of fewer medications.[5]

Self-care is defined as “actions directed toward oneself or the environment to regulate one’s functioning in the interest of one’s life, integrated functioning, and well-being.” [6] The concept of self-care is multi-dimensional. The capacity for self-care depends on a person’s “self efficacy”, “motivation” to continue them and actual execution of these activities as reflected by the “behavior”. [7] A study in Beijing on self-care behaviours showed more than 70% of patients with hypertension were non-smoker and abstained from alcohol consumption.[8] However, self-care behaviours related to medication adherence, regular blood pressure monitoring, and physical exercise were less commonly reported. In Saudi Arabia, the overall self-care level was moderate with adherence to medication scored the highest in patients with hypertension.[9] The associations between level of self-care behaviour and age, gender, education status and duration of having hypertension were reported in both studies.[8,9]

Studies have shown that self-care behaviour among hypertensive patients correlate with blood pressure control and prevents complications of hypertension.[3,10] Motivation and self-efficacy were shown to be associated with improved medication adherence and lifestyle modifications.[11,12]

There are tools that evaluate the level of self-care among patients with hypertension. A systematic review on 29 articles that assessed hypertension self-care assessment instruments revealed deficiencies in some of these instruments.[13] Most of these instruments only emphasized on behaviour related to adherence to medications, based on dissimilar theoretical framework and poor psychometric quality.[13] The Hypertension Self-Care Profile (HTN SCP) is a comprehensive tool, which encompasses three domains that assess behaviour, motivation and self-efficacy in patients with hypertension.[7]

Self-care had positive effects on clinical outcomes of hypertension such as reduction in the occurrences of stroke and premature cardiac deaths.[3,10,14,15] However to date, there is no published data on the multiple dimensions of hypertension self-care in Malaysia with the use of Hypertension Self-Care Profile (HTN-SCP) tool. The HTN-SCP tool was validated in Singapore with a similar multi-ethnic Asian population as in Malaysia. [7,16,17,18] It provides opportunities for cross-national comparison of hypertension self-care capacity in different populations. In addition, the tool allows profiling patients using the three dimensions of self-care in hypertension. Thus, this study aimed to determine the self-care profiles of patients with hypertension in the primary care clinics using the comprehensive Hypertension Self-Care Profile (HTN-SCP) questionnaire. [7] We hypothesized that the level of self-care for managing hypertension among Malaysian adults is low. As alluded in the Beijing and Saudi Arabic studies [8,9] there will be significant associations between self-care with socio-demographic and hypertension-related factors. The findings from this study will provide information to initiate future efforts to promote self-care among patients with hypertension and strengthen the delivery of self-care education among healthcare professionals.

## Methods

This cross sectional study was conducted between 1 October 2016–30 April 2017 in 3 public primary care clinics in the state of Selangor, Malaysia. These clinics were selected using a simple random sampling of all public primary care clinics in the whole state of Selangor. These clinics were located at the urban areas with a high attendance of hypertensive patients. Besides, these clinics were government-funded clinics accessible to the whole population at a minimal cost. All adults aged 18 years and above with hypertension for at least 6 months, who understands and able to answer the questionnaire in English, Chinese and Malay language were included. The exclusion criteria for the study were pregnant women, those with acute psychotic illness or cognitive impairment. The sample size was calculated using the Lemeshow and Lwanga(1990) [19] formula based on a study by Neminqani et al (2013)[9] using the mean of self-management score among those aged <60 years and ≥60 years with the confidence level of 95%, and power of 90%. The total sample size was 720, after taking into consideration a 20% drop-out and missing data.

### Study instrument

The participants were assisted in the administration of the study instrument in either English, Chinese or Malay language. The study instrument comprises three parts:

#### Part one: Socio-demographic information

1. Age
2. Gender
3. Ethnic
4. Marital status
5. Household income
6. Education level

#### Part two: Medical information

1. Duration diagnosed with hypertension
2. Recent blood pressure reading
3. Weight and height: to calculate body mass index
4. Number anti-hypertensive medications
5. Family history of hypertension
6. Other medical problems

The medical information was verified using the participant’s medical record and was taken from the most recently entered information.

#### Part three: The Hypertension Self-Care Profile (HTN-SCP) tool (English version).[14]

The HTN SCP tool[14]: It is a self-administered English questionnaire, consisting of 60 items grouped into three sections–Motivation, Behaviour and Self-efficacy, with each section has 20 items. This tool has been validated and it is a reliable tool. The questionnaire validation has been done by the Singapore collaborator study team. [7,14,15,16] The Cronbach’s Alpha for motivation, behaviour and self-efficacy were 0.948, 0.857 and 0.931, respectively.The interclass correlation co-efficients for motivation, behaviour and self-efficacy were 0.762, 0.671 and 0.720, respectively. The Cronbach’s Alpha for motivation, behaviour and self-efficacy were 0.928, 0.851 and 0.945 for the Malay version and 0.838, 0.929, and 0.927 for the Chinese version, respectively. [15,16] There are three sections for the HTN SCP questionnaire. The questions in each section seek information on the patient’s physical activity, recommended dietary intake, habits of reading nutritional facts and labels, restriction of alcohol consumption, smoking habit, home blood pressure monitoring, compliance to medication and follow up and weight reduction. For each section, the questions commence with the following stems: (1) how often do you do (behavior); (2) how important you think it is to do (motivation); and (3) how confident are you in doing a particular task (self eficacy)? Each section comprised of 20 questions. Each question uses a 4-point ordinal scale. For each section, the higher scores indicates higher level of hypertension self-care behaviour, motivations and self efficacy. For each section, the minimum score is 0 and the maximum score is 60.

The questionnaire was also pre-tested on 30 local participants prior to the study. Minor amendments were made after the pilot study, such as inclusion of locally available food types for illustration in the questionnaire.

The target for blood pressure control was based on the Malaysian Hypertension and Diabetes management guidelines, [20,21] with blood pressure level of <140/90 mmHg for non-diabetic, and ≤ 135/75 mmHg for those with diabetes or chronic kidney disease. The Body Mass Index (BMI) classification was according to the Malaysian obesity guideline [22]: BMI of Underweight <18.5 kg/m^2^, Normal weight 18.5–22.9 kg/m^2^, Overweight 23–27.4 kg/m^2^ and Obese > 27.5 kg/m^2^.

### Data collection

Patients were recruited between 1 October 2016–30 April 2017 at the primary care clinics using systematic random sampling with the sampling interval of two. The first patient recruited on day one of data collection was randomly selected by drawing a lot and became the reference point of the study recruitment. Subsequently every alternate patient was approached for recruitment.

Patients who agreed to participate were informed about the study and were provided informed consent. A pretested proforma contained baseline sociodemographic data were self-administered by the patients where as HTN SCP were interviewer-administered. The clinical information of the patients were obtained from their medical records.

### Data analysis

Statistical analysis was done using SPSS version 20.0. Descriptive statistics were used to describe demographic and disease characteristics of the patients and their blood pressure control. Percentages and frequencies were used for the categorical variables while mean and standard deviations were calculated for the continuous variables. Normality testing was done for numerical data using the calculation of z-score from skewness and kurtosis, Kolmogorov-Smirnov, histogram and Q-Q plot, which showed data was normally distributed. The association for numerical data were tested using t-test and One way ANOVA. Multiple linear regressions (MLR) were performed to identify the determinants for hypertension self-care as the HTN-SCP score is a continuous variable. Furthermore, there is no cut off point to categorise good or poor self-care in hypertension in the original version of HTC-SCP questionnaire. Univariate analyses using independent t-tests and One-way ANOVA analysis.were performed and factors with p<0.25 were included in the MLR model. The results of the MLR were presented as beta coefficient, standard error and 95% confidence intervals. Significant level was set at p<0.05. The assumptions of multiple linear regression analysis was met. Firstlly, the scatterplots showed linear relationship between the dependent and independent variables. Secondly, the histogram and goodness of fit test showed normal distribution of residue values. Thirdly, there were no multcollinearity between the independent variables as the tolerance values were above 0.1 (ranging from 0.865 to 0.987) and the variance inflation factors (VIF) were less than 3 (ranging from 1.013 to 1.148). Lastly, a scatterplot of residuals versus predicted values showed no clear pattern in distribution (homoscedasticity).

### Ethics approval

Ethical approval was obtained from the Medical Research & Ethics Committee of the Ministry of Health Malaysia: NMRR-17-1508-36071. Subsequently permission to have access to the electronic medical record at the public primary care clinics was sought from the Selangor state Health Department since these three clinics was under their jurisdiction. A briefing session was held at the selected clinics. All medical officers, specialists and medical staff were informed and explained regarding the study. Participants were explained about the study and written consent were obtained prior to their participation in this study. The participants were assigned non-identifiable identification codes for data entry and data analysis. All the consent forms and questionnaires are stored in a locked filing cabinet accessible only by the researchers and will be stored for 7 years. After this time information will be shredded and disposed of in secure bins. The university IT department will assist in deleting the data from storage after the completion of the project.

## Results

We approached 811 patients and 50 of them refused to participate due to time factor and language barrier. Hence, the response rate was 93.8%. For those who agreed to participate, 31 (3.8%) of the questionnaires were excluded from analysis due to incomplete data. The final number of patients’ data included for analysis was 730. The mean age of the patients was 59.5 (SD 10.23) years old with 52.5% of them aged 60 years and above. There were more women (52.5%) and most of the participants were Malays (44.0%), followed by Chinese (34.9%) and Indians (21.1%). More than half (58.4%) of them had secondary school level of education, followed by 24.8% with primary or no formal school and 6.8% with tertiary level of education. Majority of them were married (81.5%) and from the lower socio-economy population (71.2%) with monthly household income of less than RM3000.

Table 1 shows the disease characteristic of the participants. Majority of them had hypertension for 5 years or above (69.6%) and had other co-morbidities (81.4%). Approximately one third (30.7%) of them had family history of hypertension. About half (56%) of them were on two or more anti-hypertensive medications. However, most (65.5%) of them did not have their blood pressure controlled to the targets. Majority of the patients were either overweight (32.9%) or obese (48.6%) and more than half (53%) of them had diabetes mellitus or chronic renal disease. (Table 1)

**Table 1:** Disease characteristic of the study participants (N = 730).

Variable	Result N(%)	Mean (SD)
**Duration of Hypertension (years)**		9.6(7.9)
< 5	222 (30.4)	
> 5	508 (69.6)	
**Family History of Hypertension**		
No	506 (69.3)	
Yes	224 (30.7)	
**Other medical Conditions**		
Yes	594 (81.4)	
No	139 (18.6)	
**No. of anti-hypertensive Medications**		
0	7(1.0)	
1	312(42.7)	
2	259(35.5)	
3	117(16.0)	
≥4	35 (4.8)	
**Blood pressure controlled (Non DM < 140/90, DM/CKD < or = 135/75)**		
No	478 (65.5)	
Yes	252 (34.5)	
**Body Mass Index (BMI) kg/m**^**2**^		
Underweight (<18.5)	8 (1.1)	
Normal weight (18.5–22.9)	119 (16.3)	
Overweight (23–27.4)	240 (32.9)	
Obese (≥ 27.5)	355 (48.6)	
Missing data	8 (1.1)	
**Present of Diabetes/CKD**		
Yes	387(53.0)	
No	343(47.0)	

Table 2 shows the scores of the respective domains in the HTN SCP tool. The mean total score of hypertension self-care was 124.2 (SD 22.8) with minimum score of 42 and maximum score of 178. The mean scores were highest for motivation (mean 45.3, SD 9.1), followed by self-efficacy (mean 42.5, SD 8.9) and lowest for behaviour (mean 36.4, SD 8.9).

**Table 2:** Scores of Hypertension Self-Care Profiles.

DOMAIN	MINIMUM SCORES	MAXIMUM SCORES	MEAN SCORES	Standard deviation
Behavior	11	59	36.4	8.9
Motivation	3	60	45.3	9.1
Self-efficacy	11	60	42.5	8.9
**Total Hypertension Self-Care Profiles score**	**42**	**178**	**124.2**	**22.8**

Table 3 shows the associations between Total Hypertension Self-Care Profile score and sociodemographic factors and disease characteristic using univariate analyses. Women (mean 126.0, SD 22.3) were found to have significant higher score (P-value 0.018) compare to men (mean 122.0,SD 23.4). For the different ethnic groups, Indians (mean 130.5, SD 21.7) and Malays (mean 128.1, SD 19.8) were found to have significantly higher scores (P-value <0.001) than Chinese (mean 115.1, SD24.2). Those with Diploma or tertiary education (mean 133.3, SD23.6) had significantly higher scores (P-value <0.001) than those with secondary school (mean 125.3, SD 22.5) and primary school level education (mean 118.9, SD 21.7). Participants with family history of hypertension (mean 125.9, SD 22.3) scored higher (P-value 0.002) than those without (mean 120.3, SD 23.7). The Total Hypertension Self-Care Profile score was not significantly associated with age, household income, marital status, duration of hypertension, presence of other medical conditions, number of anti-hypertensive medication, blood pressure control, body mass index and presence of diabetes or chronic renal disease.

**Table 3:** Associations between Hypertension Self-Care Profile scores and sociodemographic factors and disease characteristic.

Variable	Total Hypertension Self-Care Profile score Mean+/-SD	P-value
**Age,years**^**a**^		0.833
<60	124.0 (22.6)	
≥60	124.4 (23.1)	
**Gender**^**a**^		0.018*
Women	126.0 (22.3)	
Men	122.0 (23.4)	
**Race**^**b**^		<0.001*
Malay	128.1 (19.8)	
Chinese	115.1(24.2)	
Indian	130.5(21.7)	
**Education level**^**b**^		<0.001*
No formal education/Primary school	118.9 (21.7)	
Secondry school	125.3 (22.5)	
Diploma/Tertiary	133.3 (23.6)	
**Household income (per month)**^**a**^		0.672
<RM 3000	124.0 (22.5)	
≥RM 3000	124.8 (23.8)	
**Marital status**^**a**^		0.053
Married	124.5(22.6)	
Single/separated/widowed	123.8(23.9)	
**Duration of hypertension (years)**^**a**^		0.819
< 5	124.5 (22.8)	
≥ 5	124.1(22.9)	
**Family History of** **hypertension**^**a**^		0.002*
No	120.3 (23.7)	
Yes	125.9 (22.3)	
**Other medical conditions**^**a**^		0.066
Yes	123.5 (22.5)	
No	127.4 (24.5)	
**No.of anti-hypertensive medications**^**b**^		0.946
0	121.7 (14.0)	
1	124.6 (22.8)	
2	124.4 (23.5)	
3	122.7 (22.6)	
≥ 4	125.1 (21.1)	
**Blood pressure controlled** **(Non DM < 140/90mmHg,** **DM/CKD < or = 135/75mmHg)**^**q**^		0.798
No	124.0(23.3)	
Yes	124.5 (21.5)	
**Body Mass Index (kg/m**^**2**^**)**^**b**^		0.198
Underweight (<18.5)	124.4 (10.7)	
Normal weight (18.5–22.9)	121.9 (24.0)	
Overweight (23–27.4)	122.4 (23.1)	
Obese (≥ 27.5)	125.9 (22.3)	
**Present of Diabetes/CKD**^**a**^		0.289
Yes	123.4 (23.0)	
No	125.2 (22.7)	

Univariate analysis (t-test^a^ or

One-way ANOVA^b^).

* P-value < 0.05.

The significant determinants that influenced the Total Hypertension Self-Care Profile scores from the multiple linear regression model were gender, ethnicity, education level and family history of hypertension. Men had lower self-care profile scores than women (B -4.5, P-value 0.008, 95% Cl -7.8 to -1.2). The ethnic group Chinese scored lower than Indian and Malay (B -14.7, P-value <0.001, 95% CI -19.0 to -10.3) Those with lower education level (primary level education/no formal school: B -15.7, P-value <0.001, 95% CI -21.0 to -10.4 and secondary level education: B -9.2, P- value <0.001, 95% CI -14.0 to -4.4) had lower scores than those with tertiary level education. Those with family history of hypertension (B 4.4, P-value 0.014, 95% CI 0.9 to 7.8) had higher scores than those without. (Table 4)

**Table 4:** Multiple linear regression of Total Hypertension Self-Care Profile score and the associated sociodemographic and disease characteristic factors.

Factors	B	SE	t	p-value	95% Cl	
					Lower	Upper
**Gender**						
Female	Ref	**-**	**-**	**-**	**-**	**-**
Male	-4.5	1.7	-2.641	**0.0008**	-7.8	-1.2
**Race**						
Indian	Ref	-	-	-	-	-
Malay	-3.5	2.1	-1.652	**0.099**	-7.6	0.7
Chinese	-14.7	2.2	-6.624	**<0.001**	-19.0	-10.3
**Education level**						
Tertiary	Ref	-	-	-	-	-
Secondry school	-9.2	2.4	-3.798	**<0.001**	-14.0	-4.4
No formal education/ Primary school	-15.7	2.7	-5.845	**<0.001**	-21.0	-10.4
**Marital status**						
Single/searated/ Widowed	Ref	-	-	-	-	-
Married	1.9	2.2	0.884	0.377	-2.3	6.1
**Family History of hypertension**						
No	Ref	-	-	-	-	-
Yes	4.4	1.8	2.460	**0.014**	0.9	7.8
**Other medical conditions**						
Yes	Ref	-	-	-	-	-
No	-3.6	2.1	-1.734	0.083	-7.7	0.5
**Body Mass Index (BMI) kg/m**^**2**^						
Obese (> 27.5)	Ref	-	-	-	-	-
Overweight (23–27.4)	-2.2	1.9	-0.385	0.245	-5.8	1.5
Normal weight (18.5–22.9)	-3.0	2.4	-1.233	0.218	-7.7	1.8
Underweight (<18.5)	-3.0	7.7	-1.164	0.700	-18.1	12.2

Bold = P-value <0.05, SE = standard error, CI = confidence interval, B = β coefficient, t = t-score of the regression model.

Adjusted R square = 0.137

## Discussion

In this study, we aimed to determine the level of self-care and the factors influencing the level of self-care among adults with hypertension in the public health care clinics in the urban setting of Selangor state, Malaysia. Overall, this study found that the mean Total Hypertension Self-Care Profile (HTN-SCP) score was 124.19 (SD 22.84). Factors that influenced the Total HTN-SCP score were gender; men score lower than women, ethnic group; Chinese score lower than Malays and Indians, education level; patients with primary level education or no formal school and secondary level education score lower than those with tertiary level education. Family history of hypertension was associated with higher score than those without. The hypertension population in this study comprised of 44% Malays, 34% Chinese and 21% Indians. The baseline characteristics of the hypertensive patients were not quite similar compared to those in the general population, with reference to the Malaysia Health and Morbidity Survey 2015 in which the prevalence of hypertension was 31.1% in Malays, 30.8% in Chinese and 32.4% in Indians. [23] We have to be aware that these were two completely different population where our study was conducted in primary care clinic setting whereas the Malaysia Health and Morbidity Survey 2015 was conducted in the general population.

The mean Total HTN-SCP score in this study was 124.19 (SD 22.84) out of the maximum score of 180, which was about 68.9% of the total maximum score. This finding suggested moderate level of hypertension self-care in this study population. The lowest score (mean 36.43, SD 8.92) was identified in the behaviour scale, which attained only 60.7% out of the maximum score of 60.

In this study, men were found to have lower hypertension self-care score than female. This was consistent with other studies which reported higher level of self care among women as compared to men. [8,9,24] Men might have other priorities in life instead of their health, which could have contributed to their poorer health outcomes compared to women.[25]

It was unexpected to find that Chinese had lower Total HTN-SCP score compared to Malays and Indians in this study. Other studies have reported better health-seeking behavior and self-care behavior among Chinese compared to other ethnic groups. [26–27] One possible reason the differences observed could be related to language barrier. In Malaysia health facilities, most health information materials were in English or Malay languages, which may hinder the self-efficacy of Chinese patients in managing their hypertension. Studies had reported that health literacy is linked to language proficiency.[28] As most of the patients in our study have lower education level, they may have difficulty in understanding health information that were delivered in other than their native languages. Further studies may be needed to explore other factors leading to poorer self-care behaviour of this minority ethnic group.

Higher education level was associated with higher self-care scores in this study. This is consistent with another study [9] as higher education was correlated with increased health literacy, leading to better understanding of the health information materials and advice by the healthcare professionals.

A positive family history of hypertension was associated with higher hypertension self-care score. In contrast, most studies did not report similar finding. [29,30] Asian families are close-knitted in Malaysia, and parents are likely to be looked after by their children living within their households. The interaction between patients with hypertension and other family members could have mutually influenced their awareness and behaviour towards self-care measures in hypertension. The influence of socio-cultural factors and Asian family dynamics should be further explored in future studies.

The study highlights the need for the development of self-care intervention for patients with hypertension. Taylor et. al. (2014) reviewed the evidence on interventions supporting self-care for people with long term conditions [31] Among people with hypertension, interventions to promote self-care covered strategies such as technological intervention [32], lifestyle change [33], behavioural contracts between patients and healthcare professionals [34], simplified medication regime [35], reminders for appointments [36] and complex, professional-led care. [37] In these interventions, technology has shown positive results as a standalone intervention in terms of BP control as compared to others. [31] Evidence has also suggested that interventions, which combined lifestyle change and simplified drug regime improved self care. [31] Thus, effective interventions to elevate the self-care profile of patients with hypertension are likely to be multi-faceted and involve partnership with the healthcare professionals. [32] Such complex interventions will be tested for effectiveness in future studies in Malaysia. Earlier studies did not allow for the evaluations of the various dimensions of self-care in hypertension. We believe this study provides the evidence that the capacity for self-care is quantifiable using a validated tool. The baseline data allows comparison in future interventional studies and across different populations with hypertension. While we have yet to determine the cut-off to distinguish patients with different capacities for self-care, a cohort study is in the pipeline to substantiate the scientific evidence of the determinants and health outcomes.

### Strengths and limitations

This was the first study to comprehensively describe self-care motivation, self-efficacy and self-care behavior of hypertensive patients in a multi-ethnic Southeast Asian country, of which data are limited in this area. The findings provide useful insights into the level of self-efficacy and self-care of Malaysians with hypertension.

However, there were several limitations in this study. Firstly, as this was a cross-sectional study, causality could not be determined. Secondly, we understand that a randomized selected study population would be ideal. However, this was not feasible in the study setting due to its appointment-based system, in which the investigators had no means of controlling the appointment booking of the patients. Furthermore, there is no patient registry at the study site. In this study, the systematic random sampling method was used for patient recruitment in view of its feasibility, the fact that the spreads of the patients were more evenly throughout the population and most importantly, it stills classified under probability sampling method. Thus, the finding can still be generalizable to the other public primary health care clinics in the urban setting but this may not represent the population in rural area and other clinical setting. Thirdly, recall bias may be inevitable as the data in this study were obtained through a questionnaire. The Hawthorne effect among the participants cannot be excluded as patients may report socially acceptable behaviour which cannot be authenticated. Nevertheless, the results were not skewed as the average scores hovered around the mid-range. Lastly, the low adjusted R-squared in this study may indicate low percentage of the response variable variation that is explained by the linear model. However, low R-squared can still provide a useful clinical model with respect to data trends, but may be low in precision.[38] Despite the lack of studies using the HTN-SCP tool, it has been validated and contextualized in a multi-ethnic Asian population. [14,15,16] Future studies will look into comparison of different population with hypertension using the HTN- SCP.

## Conclusion

The overall hypertension self-care profile among patients in this multi-ethnic country was moderate. Being men, Chinese, of lower education level and without family history of hypertension were associated with lower hypertension self-care profile based on the HTN-SCP score. Healthcare intervention programmes to address self-care in hypertension should be implemented to all population but more attention should be given to patients fitting these characteristics of lower self-care profile.

## References

[1] WHO | World Health Day 2013. World Health Organization Global Brief on Hypertension. Available from: http://www.who.int/campaigns/world-health-day/2013/en/ Cited 12 April 2016.

[2] Institute for Public Health. National Health and Morbidity Survey (NHMS) 2015;2:17–9. Available from: http://www.iku.gov.my/index.php/research-eng/list-of-research-eng/iku-eng/nhms-eng/nhms-2015 Cited 4 September 2018.

[3] Fan AZ, Mallawaarachchi DSV, Gilbertz D, Li Y, Mokdad AH. Lifestyle behaviors and receipt of preventive health care services among hypertensive Americans aged 45 years or older in 2007. Prev Med (Baltim). 2010;50(3):138–142. 10.1016/j.ypmed.2009.12.007 20006640

[4] Lin P-H, Appel LJ, Funk K, Craddick S, Chen C, Elmer P, et al The PREMIER intervention helps participants follow the Dietary Approaches to Stop Hypertension dietary pattern and the current Dietary Reference Intakes recommendations. J Am Diet Assoc. 2007;107(9):1541–1551. 10.1016/j.jada.2007.06.019 17761231

[5] Bodenheimer T. Patient Self-management of Chronic Disease in Primary Care. JAMA. American Medical Association. 2002;288(19):2469 10.1001/jama.288.19.2469 12435261

[6] Orem DE. Concepts of practice 3 New York: McGraw-Hill; 1985

[7] Han H-R, Lee H, Commodore-Mensah Y, Kim M. Development and validation of the Hypertension Self-care Profile: a practical tool to measure hypertension self-care. J Cardiovasc Nurs. 2014;29(3):E11–20. 10.1097/JCN.0b013e3182a3fd46 24088621 PMC3972381

[8] Hu H, Li G, Arao T. Prevalence rates of self-care behaviors and related factors in a rural hypertension population: a questionnaire survey. Int J Hypertens. 2013;2013:526949 10.1155/2013/526949 23819042 PMC3683479

[9] Neminqani DM, El-Shereef EAA, Thubiany MMAL. Hypertensive Patients: Self-Care Management Practices in Al-Taif, KSA. Int J Sci Res. 2013; 12(4): 1705–1714.

[10] Schroeder K, Fahey T, Ebrahim S. How can we improve adherence to blood pressure-lowering medication in ambulatory care? Systematic review of randomized controlled trials. Arch Intern Med. 2004;164(7):722–732. 10.1001/archinte.164.7.722 15078641

[11] Britt E, Hudson SM, Blampied NM. Motivational interviewing in health settings: a review. Patient Educ Couns. 2004;53(2):147–155. 10.1016/S0738-3991(03)00141-1 15140454

[12] Resnick B, Shaughnessy M, Galik E, Scheve A, Fitten R, Morrison T, et al Pilot testing of the PRAISEDD intervention among African American and low-income older adults. J Cardiovasc Nurs. 2009;24(5):352–61. 10.1097/JCN.0b013e3181ac0301 19652618

[13] Han H-R, Song H-J, Nguyen T, Kim MT. Measuring self-care in patients with hypertension: a systematic review of literature. J Cardiovasc Nurs. 2014;29(1):55–67. 10.1097/JCN.0b013e3182775fd1 23348221

[14] Dickinson HO, Mason JM, Nicolson DJ, Campbell F, Beyer FR, Cook J V, et al Lifestyle interventions to reduce raised blood pressure: a systematic review of randomized controlled trials. J Hypertens. 2006;24(2):215–233. 10.1097/01.hjh.0000199800.72563.26 16508562

[15] Warren-Findlow J, Seymour RB, Brunner Huber LR. The association between self-efficacy and hypertension self-care activities among African American adults. J Community Health. 2012;37(1):15–24. 10.1007/s10900-011-9410-6 21547409 PMC3179559

[16] Koh YLE, Lua YHA, Hong L, Bong HSS, Yeo LSJ, Tsang LPM, et al Using a Web-Based Approach to Assess Test-Retest Reliability of the “Hypertension Self-Care Profile” Tool in an Asian Population: A Validation Study. Medicine (Baltimore). Wolters Kluwer Health; 2016;95(9):e2955. 10.1097/MD.0000000000002955 PMC4782894 26945410

[17] Seow KC, Mohamed Yusoff D, Koh YLE, Tan NC. What is the test-retest reliability of the Malay version of the Hypertension Self-Care Profile self efficacy assessment tool? A validation study in primary care. BMJ Open. 2017;7(9):e016152 10.1136/bmjopen-2017-016152 28882912 PMC5588997

[18] Ngoh SHA, Lim HWL, Koh YLE, Tan NC. Test-retest reliability of the Mandarin versions of the Hypertension Self-Care Profile instrument. Medicine (Baltimore). 2017;96(45):e8568 10.1097/MD.0000000000008568 29137076 PMC5690769

[19] Lemeshow, Stanley, Hosmer, David W, Klar, Janelle, Lwanga et al. Adequacy of sample size in health studies 1990. Available from: http://www.who.int/iris/handle/10665/41607. Cited 4 April 2016

[20] Clinical practice guidelines, Management of Diabetes Mellitus. Ministry of Health Malaysia; 2015. Available from: http://www.moh.gov.my/penerbitan/CPG/CPG%20T2DM%202015.pdf. Cited 4 April 2016.

[21] Clinical practice guidelines, Management of Hypertension. Ministry of Health Malaysia; 2013.

[22] Clinical practice guidelines, Management of Obesity. Ministry of Health Malaysia; 2004.

[23] Institute for Public Health (IPH) 2015. National Health and Morbidity Survey 2015 (NHMS 2015) Ministry of Health, Malaysia.

[24] Motlagh Sayed Fazel Zinat, Chaman Reza, Sadeghi Erfan, and Eslami Ahmad Ali. Self-Care Behaviors and Related Factors in Hypertensive Patients. Iran Red Crescent Med J. 2016; 18(6):e35805. 10.5812/ircmj.35805 PMC5004506 27621938

[25] WHO. The men’s health gap: men must be included in the global health equity agenda. Available from: http://www.who.int/bulletin/volumes/92/8/13-132795/en/ Cited 4 September 2018.

[26] Tan Andrew K.G., Dunn Richard A., and Yen Steven T. Ethnic Disparities in Metabolic Syndrome in Malaysia:An Analysis by Risk Factors. Metab Syndr Relat Disord. 2011;9(6):441–451 10.1089/met.2011.0031 21815810 PMC3225058

[27] Choong Y O, Tan C E, Lim Y H, Tan M Z & Liew C Y. Help seeking behaviour among secondary school students in Malaysia. E-Proceeding of the International Conference on Social Science Research, ICSSR 2015 (e-ISBN 978-967-0792-04-0). 8 & 9 June 2015, Kuala Lumpur, Malaysia.

[28] Andrulis Dennis P. and Brach Cindy. Integrating Literacy, Culture, and Language to Improve Health Care Quality for Diverse Populations. Am J Health Behav. 2007; 31(Suppl 1): S122–S133. 10.5555/ajhb.2007.31.supp.S122 17931131 PMC5091931

[29] Etesamifard Tahereh, Jouybari Touraj Ahmadi, Zinat-Motlagh Fazel, Mahbobi Mohammad, Aghaei Abba, Ataee Mari. Is the Health Belief Model as an Appropriate Predictor of the Self-Care Behaviors in Type II Diabetic Patients? Journal of Biology and today’s world 2014; 3 (12): 275–279.

[30] Tol Azar, Shojaeezadeh Davoud, Sharifirad Golamreza, Eslami Ahmadali, Mohajeritehrani Mohamadreza, Abdolvahab Baghbanian Evaluation of self-care practices and relative components among type 2 diabetic patients. J Edu Health Promot. 2012:1:19. 10.4103/2277-9531.99219 PMC3577380 23555122

[31] Taylor SJC, Pinnock H, Epiphaniou E, Pearce G, Parke HL, Schwappach A, et al A rapid synthesis of the evidence on interventions supporting self-management for people with long-term conditions: PRISMS–Practical systematic Review of Self-Management Support for long-term conditions Southampton (UK): NIHR Journals Library; 2014 12. 25642548

[32] Verberk WJ, Kessels AG, Thien T. Telecare is a valuable tool for hypertension management, a systematic review and meta-analysis. Blood Press Monit. 2011;16(3):149–55. 10.1097/MBP.0b013e328346e092 21527847

[33] McLean G, Band R, Saunderson K, Hanlon P, Murray E, Little P, et al Digital interventions to promote self-management in adults with hypertension systematic review and meta-analysis. J Hypertens. 2016;34(4):600–12. 10.1097/HJH.0000000000000859 26845284 PMC4947544

[34] Bosch-Capblanch X, Abba K, Prictor M, Garner P. Contracts between patients and healthcare practitioners for improving patients’ adherence to treatment, prevention and health promotion activities. Cochrane Database Syst Rev. 2007;2:CD004808 10.1002/14651858.CD004808.pub3 PMC6464838 17443556

[35] Ebrahim S. Detection, adherence and control of hypertension for the prevention of stroke: a systematic review. Health Technol Assess 1998;2(11). 9789758

[36] Glynn LG, Murphy AW, Smith SM, Schroeder K, Fahey T. Interventions used to improve control of blood pressure in patients with hypertension. Cochrane Database Syst Rev 2010;3:CD005182. 10.1002/14651858.CD005182.pub4 PMC13248079 20238338

[37] Chodosh J, Morton SC, Mojica W, Maglione M, Suttorp MJ, Hilton L, et al Improving patient care. Meta-analysis: chronic disease self-management programs for older adults. Ann Intern Med. 2005;143:427–440. 10.7326/0003-4819-143-6-200509200-00007 16172441

[38] Hamilton DF, Ghert M, A. H. R. W. Simpson AHRW. Interpreting regression models in clinical outcome studies. Bone Joint Res. 2015; 4(9): 152–3. Published online 2015 Sep 1. 10.1302/2046-3758.49.2000571 26392591 PMC4678365

